# Supplementary material for: In vivo screening characterizes chromatin factor functions during normal and malignant hematopoiesis
Source: Nat Genet. 2023 Aug 14;55(9):1542–54. doi: 10.1038/s41588-023-01471-2 (PMC10484791; doi:10.1038/s41588-023-01471-2)
Supplement: Supplementary file 1 — Supplementary Figs. 1–7. [file 41588_2023_1471_MOESM1_ESM.pdf]

nature genetics

Article https://doi.org/10.1038/s41588-023-01471-2

In vivo screening characterizes chromatin factor functions during normal and malignant hematopoiesis

In the format provided by the authors and unedited

Contents

Supplementary Figures 1-7.

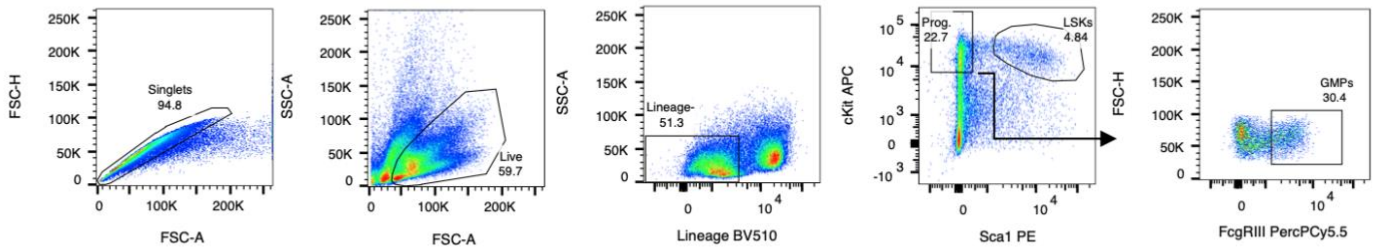

Supplementary Figure 1 | Gating strategy to isolate Haematopoietic progenitors (LSK) and Myeloid Progenitors (GMP)

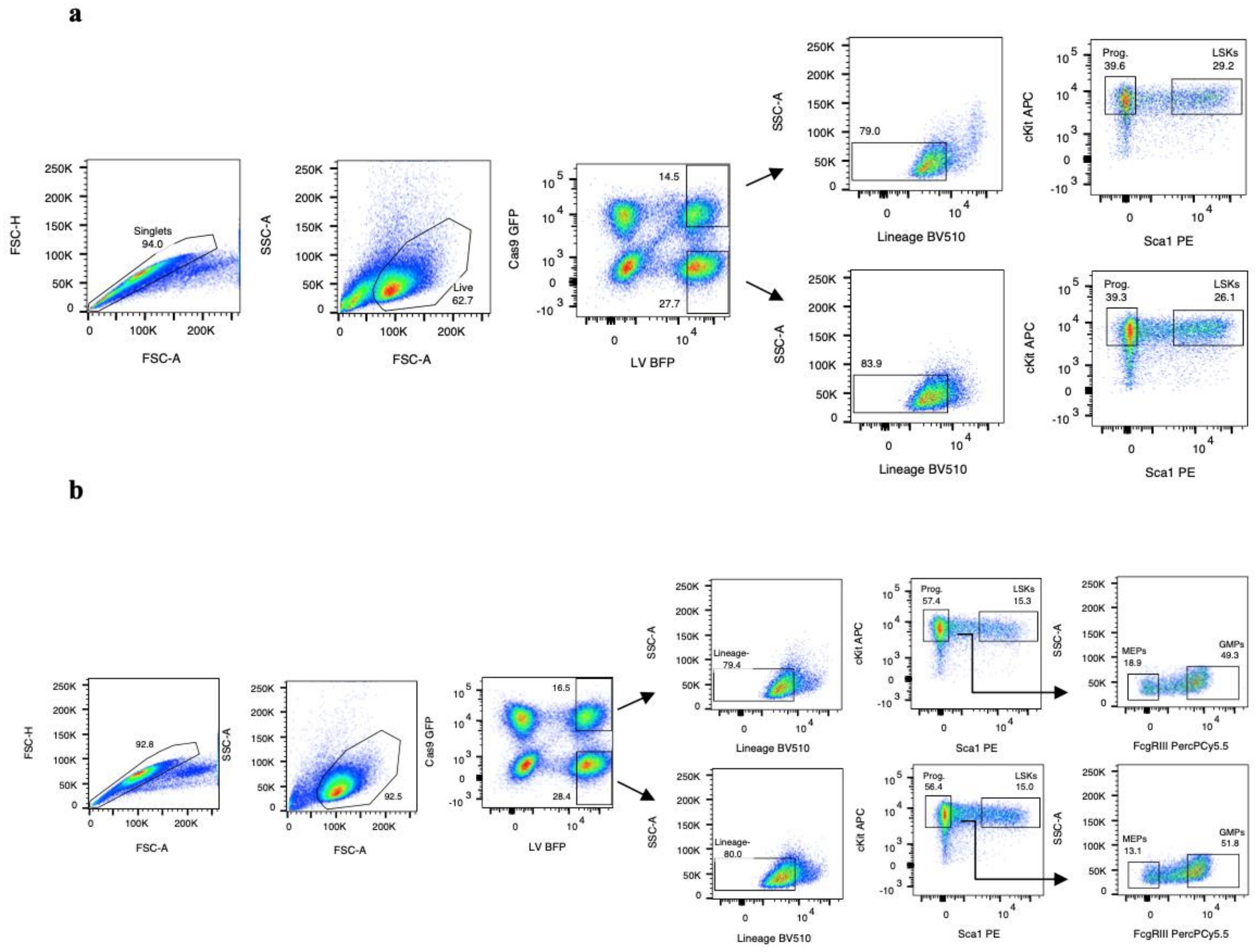

Supplementary Figure 2 | FACS Readouts for Bulk CRISPR screens. (a) Gating strategy for Self-renewal (LSK) vs Differentiation (Prog) Readout**. (b)** Gating strategy for Myeloid (GMP) vs Mega-erythroid (MEP)

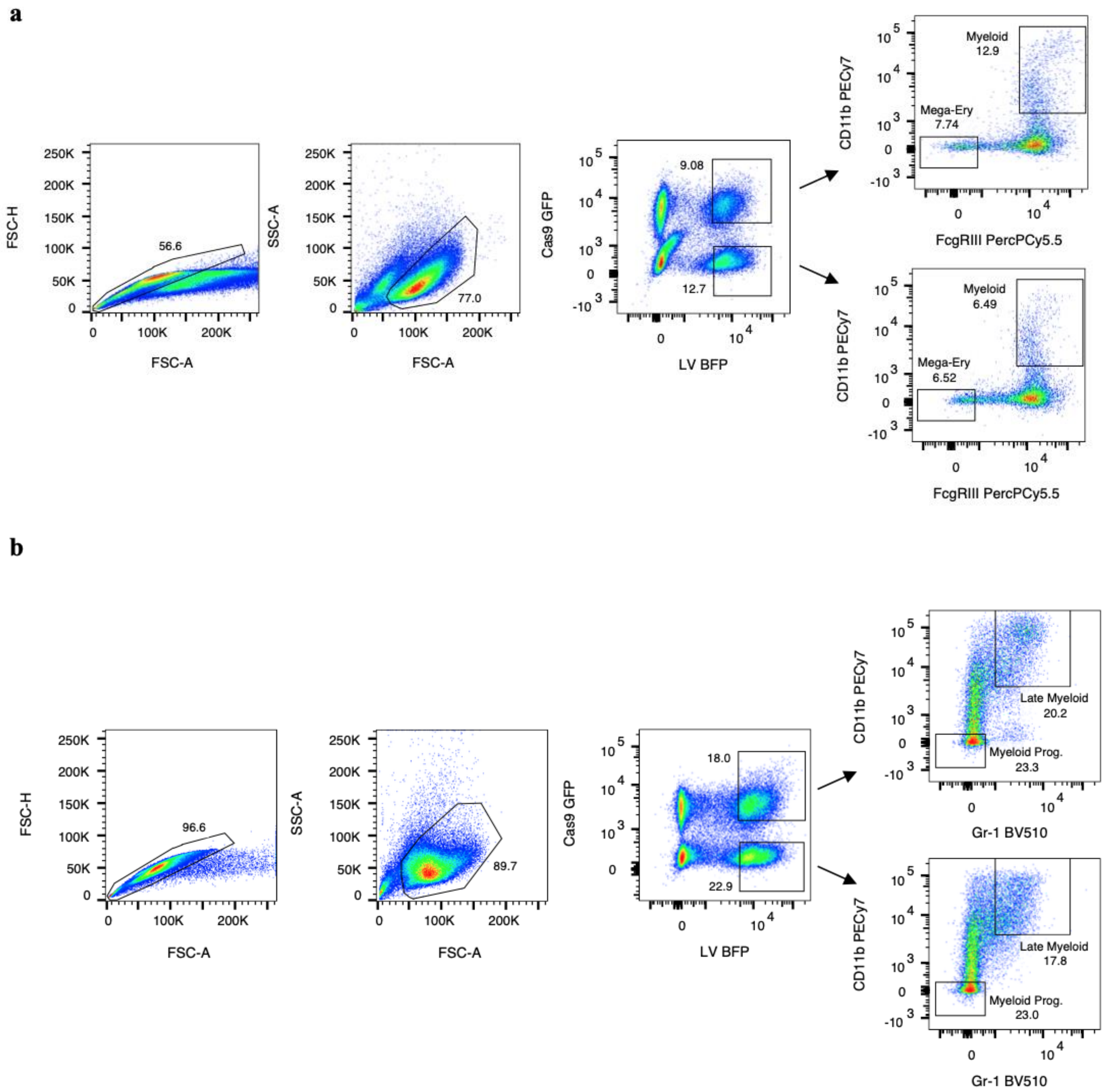

Supplementary Figure 3 | FACS Readouts for Bulk CRISPR screens. (**a**) Gating strategy for Myeloid Differentiation. (**b**) Gating strategy for Terminal Myeloid Maturation.

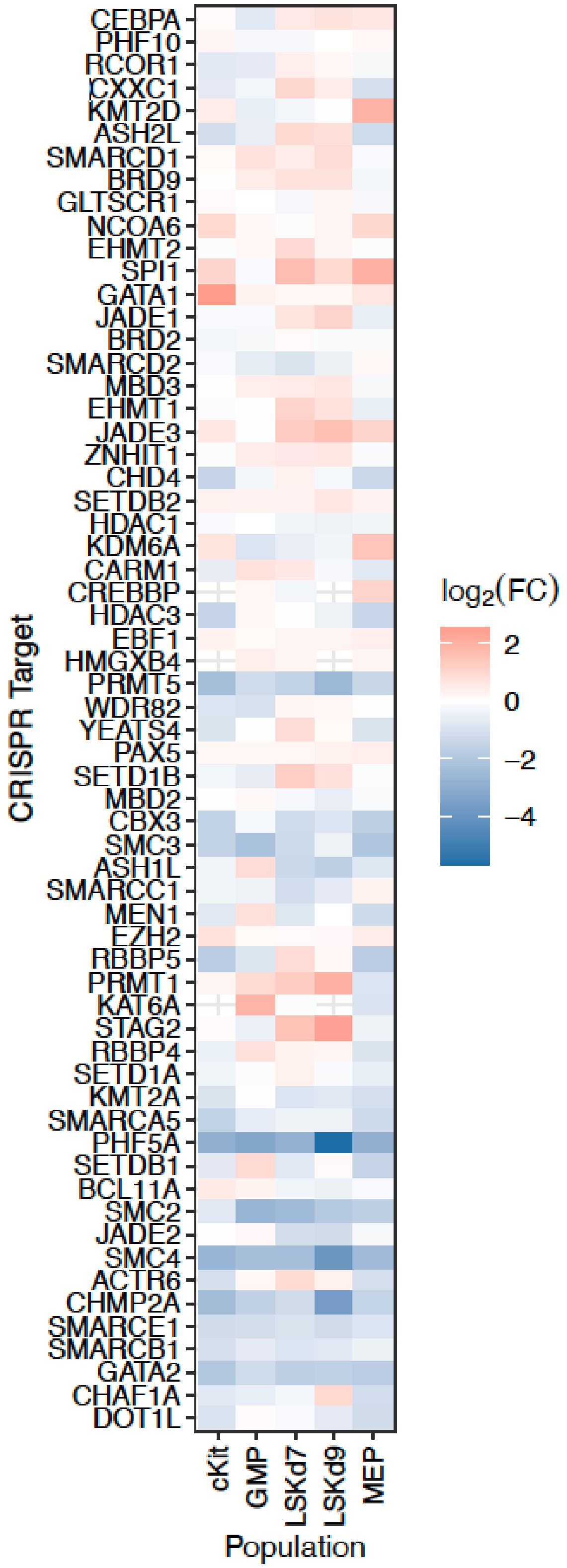

Supplementary Figure 4 | *Ex vivo* viability scores for CFs assessed in in vivo Perturb-seq The scores are calculated by comparing sgRNA abundances between Cas9 and Non-Cas9 fractions.

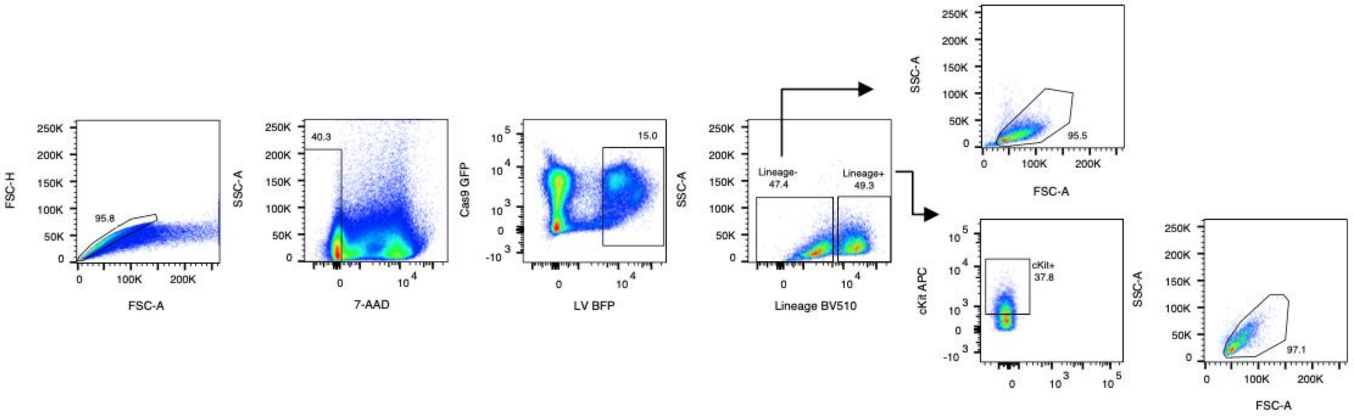

Supplementary Figure 5 | In vivo Perturb-seq gating strategy. We profiled cells derived from transplanted (GFP+), transduced (BFP+) LSK haematopoietic progenitors (gating strategy in Supplementary Figure 1) corresponding to Lineage- and Lineage+/ ckit+ gates.

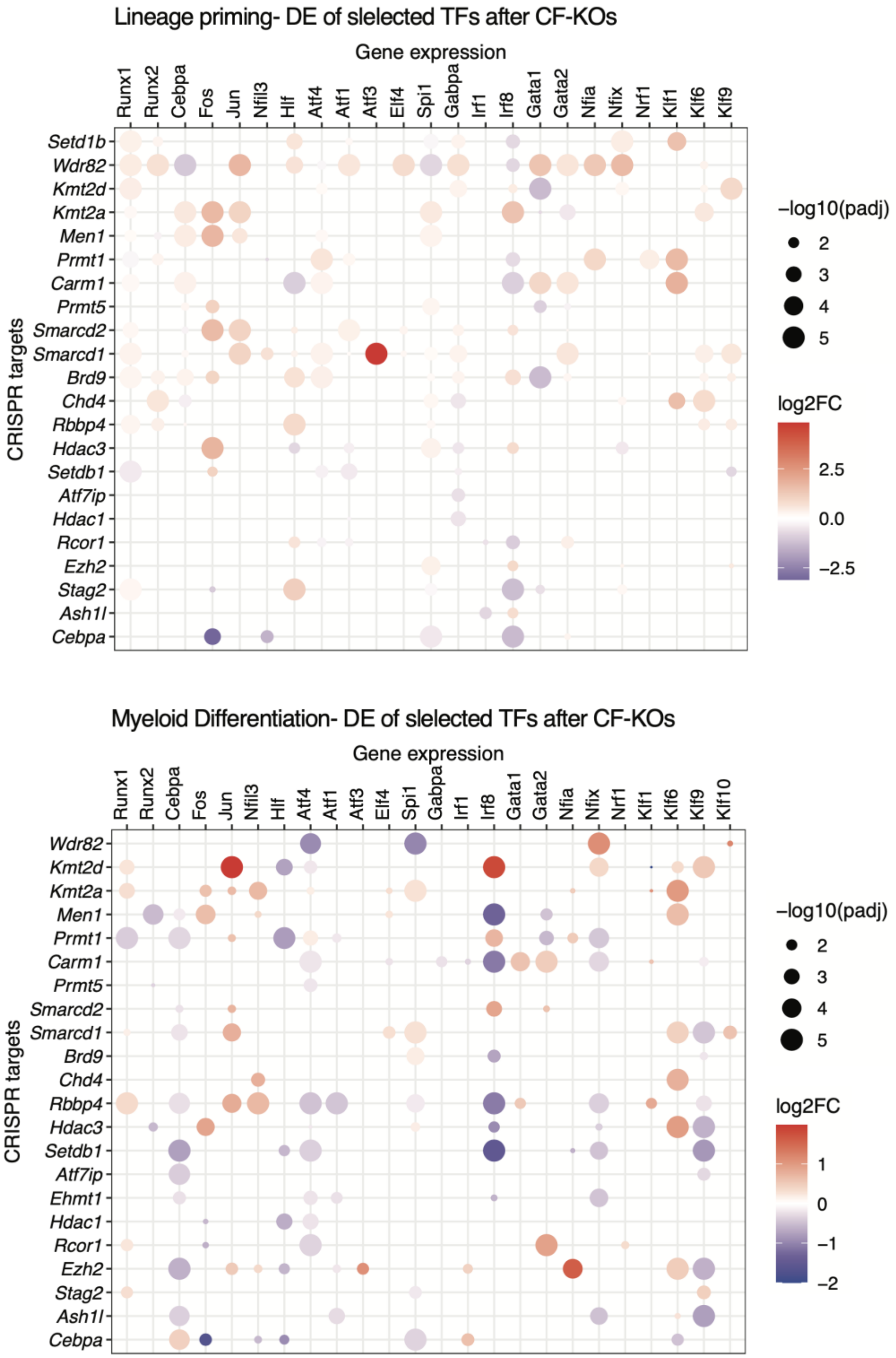

Supplementary Figure 6 |In vivo Perturb-seq. Differential expression analysis of the Transcription Factors (examined in Figure 3) after CF-KOs under ex vivo lineage priming and

myeloid differentiation conditions. P-values were calculated using negative binomial mixed models from the nebula R package.

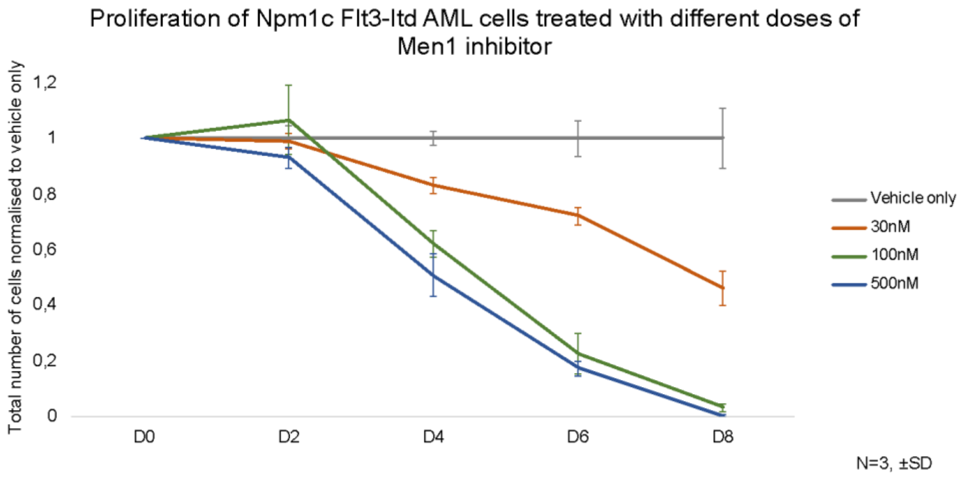

Supplementary Figure 7 | Effect of Menin inhibitor on the proliferation of *Npm1c/Flt3-ITD* leukemia cells. Cells were treated with increasing doses of the Menin Inhibitor SNDX-5613 (n=4). Error bars represent the Standard Deviation (SD).
