# Supplementary material for: In vivo screening characterizes chromatin factor functions during normal and malignant hematopoiesis
Source: Nat Genet. 2023 Aug 14;55(9):1542–54. doi: 10.1038/s41588-023-01471-2 (PMC10484791; doi:10.1038/s41588-023-01471-2)
Supplement: Supplementary file 2 — Reporting Summary [file 41588_2023_1471_MOESM2_ESM.pdf]

nature portfolio

Corresponding author(s): David Lara-Astiaso, Brian Huntly, Nikolaus Fortelny

Last updated by author(s): 22/05/2023

Reporting Summary

Nature Portfolio wishes to improve the reproducibility of the work that we publish. This form provides structure for consistency and transparency in reporting. For further information on Nature Portfolio policies, see our Editorial Policies and the Editorial Policy Checklist.

Statistics

For all statistical analyses, confirm that the following items are present in the figure legend, table legend, main text, or Methods section.

n/a	Confirmed	
☐	☒	The exact sample size (*n*) for each experimental group/condition, given as a discrete number and unit of measurement
☐	☒	A statement on whether measurements were taken from distinct samples or whether the same sample was measured repeatedly
☐	☒	The statistical test(s) used AND whether they are one- or two-sided<br>*Only common tests should be described solely by name; describe more complex techniques in the Methods section.*
☐	☒	A description of all covariates tested
☐	☒	A description of any assumptions or corrections, such as tests of normality and adjustment for multiple comparisons
☐	☒	A full description of the statistical parameters including central tendency (e.g. means) or other basic estimates (e.g. regression coefficient) AND variation (e.g. standard deviation) or associated estimates of uncertainty (e.g. confidence intervals)
☐	☒	For null hypothesis testing, the test statistic (e.g. *F*, *t*, *r*) with confidence intervals, effect sizes, degrees of freedom and *P* value noted<br>*Give P values as exact values whenever suitable.*
☒	☐	For Bayesian analysis, information on the choice of priors and Markov chain Monte Carlo settings
☒	☐	For hierarchical and complex designs, identification of the appropriate level for tests and full reporting of outcomes
☒	☐	Estimates of effect sizes (e.g. Cohen's *d*, Pearson's *r*), indicating how they were calculated

Our web collection on statistics for biologists contains articles on many of the points above.

Software and code

Policy information about availability of computer code

Data collection	No software was used for data collection
Data analysis	bcl2fastq (version 2.20)<br>bowtie2 (version 2.3.4.2)<br>SAM tools (version 1.3.1)<br>R (version 4.0.2)<br>edgeR (version 3.32.1)<br>edgeR (version 3.34.1)<br>limma (version 3.46.0)<br>FlowJo (version 10.8.0)<br>FlowJo (version 10.8.1)<br>CellRanger (version 6.1.1)<br>Seurat (version 4.0.0)<br>Monocle 3 (version 0.2.3.0)<br>CytoTRACE (version 0.3.3)<br>SingleR (version 1.4.1)<br>ProjectTILs (version 2.0.2)<br>Scanpy (version 1.9.1)<br>Trim Galore (version 0.6.6)<br>Cutadapt (version 3.4)<br>ENCODE blacklist (version 2.0)

bamCoverage (version 3.5.1)
BEDTools (version 2.27.1)
MACS (version 2.2.7.1)
HOMER (version 4.10)
featureCounts (version 2.0.1)
DESeq2 (version 1.32.0)
TOBIAS (version 0.13.2)
Prism (GraphPad software; version 9.1)
nebula (version 1.1.8)
Custom code can be found here https://github.com/csbg/tfcf

For manuscripts utilizing custom algorithms or software that are central to the research but not yet described in published literature, software must be made available to editors and reviewers. We strongly encourage code deposition in a community repository (e.g. GitHub). See the Nature Portfolio guidelines for submitting code & software for further information.

Data

Policy information about availability of data

All manuscripts must include a data availability statement. This statement should provide the following information, where applicable:

- Accession codes, unique identifiers, or web links for publicly available datasets
- A description of any restrictions on data availability
- For clinical datasets or third party data, please ensure that the statement adheres to our policy

Data and Materials Availability:
Bulk Expression patterns of hematopoietic populations: Lara-Astiaso et al, Science 2012 (52), GEO accession (GSE60103)
Single-cell expression patterns of hematopoiesis: Izzo et al, Nat Genetics 2020 (45), GEO accession (GSE124822)
Perturb-seq datasets: in vivo, ex vivo and leukemic: GEO accession (GSE213511)
Chromatin accessibility of CF-KOs: GEO accession (GSE213506)
ChIP-seq datasets of CFs in vivo, ex vivo and leukemic: GEO accession (GSE213507)

Databases used in this study:
GRCm38/mm10 reference genome assembly (GENCODE vM23/Ensembl 98)

Human research participants

Policy information about studies involving human research participants and Sex and Gender in Research.

Reporting on sex and gender	No human samples were used in the study
Population characteristics	No human samples were used in the study
Recruitment	No human samples were used in the study
Ethics oversight	No human samples were used in the study

Note that full information on the approval of the study protocol must also be provided in the manuscript.

Field-specific reporting

Please select the one below that is the best fit for your research. If you are not sure, read the appropriate sections before making your selection.

☒ Life sciences ☐ Behavioural & social sciences ☐ Ecological, evolutionary & environmental sciences

For a reference copy of the document with all sections, see nature.com/documents/nr-reporting-summary-flat.pdf

Life sciences study design

All studies must disclose on these points even when the disclosure is negative.

Sample size	No sample size calculation was used. 2 biologically independent experimental replicates were performed for ChiP-seq and ATAC-seq profiling, this is a common standard in the field.<br>For the validation of CRISPR screen hits in normal and leukemia, a minimum of 3 biologically independent replicates were used, we believe this number provides strong robustness, especially when these results validate other orthogonal findings (CRISPR screens and functions derived from binding patterns).<br>In the Perturb-seq screens, we designed our experiments to assay on average 100 cells / CF-KO, which is an established standard in the field.
Data exclusions	Perturb-seq Analysis:

Data exclusions	To avoid spurious results arising from undersampling, we removed cell clusters with less than 31 cells and genes with less than 21 reads, which represent very small clusters and lowly sampled genes. These cutoffs were based on our exploratory analysis of the data and match the criteria used in seminal studies using perturbation screens (Replogle et al Cell,185,14, July 2022)
Replication	CRISPR screens and validation of candidates:<br>- Screens were performed in 2 replicates at a 500X CRISPR library coverage<br>- Validation of the effects of hits derived from the screens was performed in replicates of at least 3 independent experiments.<br>- Validation experiments were reproducible and confirmed the patterns derived from the bulk screens<br><br>In vivo Perturb-seq.<br>- Experiments were performed in different batches with all showing similar values for donor engraftment and transduction efficiency.<br>- We analysed the unperturbed patterns (cells with Non-Targeting Control guides) across batches. Batches where cells with Non-Targeting Control guides showed different trends were discarded.<br>- For the 40 top CF regulators including members of the COMPASS, BAF NurD and Repressors presented in the main figures we performed replicate experiments, which recapitulated the initial Perturb-seq patterns.<br><br>ATAC-seq of CF-KOs: All experiments were conducted in 2 replicates. All attempts of replication were successful<br><br>Leukaemia Perturb-seq.<br>- Experiments were performed in different batches with all showing similar values for transduction efficiency<br>- We analysed the unperturbed patterns (cells with Non-Targeting Control guides) across batches. Batches where cells with Non-Targeting Control guides showed different trends were discarded.<br>- For the 20 top CF regulators including members of the COMPASS, BAF NurD and Repressors presented in the main figures we performed replicate experiments, which recapitulated the initial Perturb-seq patterns.<br><br>ChIP-seq: All experiments were conducted in 2-3 replicates except for Kmt2d, Kmt2a ChIP-seq in early progenitors (GMPs and MEPs)<br>All attempts of replication were successful except for a failed ChIP-seq for Smarcb1 in MEPs that was discarded due to having low signal-to-noise.<br><br>Growth assays in CF-KOs: All experiments were conducted in 3-4 replicates. All attempts of replication were sucessful<br><br>FACS validation of chromatin factor perturbation were performed in at least 2 replicates.
Randomization	Allocation of animals for the bulk and Perturb-seq screens was randomized (using always even numbers of males and females in each experimental condition)<br>We have used bulk and single-cell (perturb-seq) to study the roles of chromatin factors in hematopoiesis and leukaemia. Our perturbations were performed in the same cell population (hematopoietic progenitors) producing a pool of different CF mutant cells. The generation of such pool of CF mutants is already a random process, thus we don't need further randomizaton
Blinding	CF-KOs and NTC controls were analyzed side by side and the experimental groups (specific CF-KOs) were experimentally determined based on the expression of gRNAs (and not assigned a priori). Thus, as groups are defined by the data, investigators could not be blinded.

Reporting for specific materials, systems and methods

We require information from authors about some types of materials, experimental systems and methods used in many studies. Here, indicate whether each material, system or method listed is relevant to your study. If you are not sure if a list item applies to your research, read the appropriate section before selecting a response.

Materials & experimental systems

n/a	Involved in the study	
☐	☒	Antibodies
☐	☒	Eukaryotic cell lines
☒	☐	Palaeontology and archaeology
☐	☒	Animals and other organisms
☒	☐	Clinical data
☒	☐	Dual use research of concern

Methods

n/a	Involved in the study	
☐	☒	ChIP-seq
☐	☒	Flow cytometry
☒	☐	MRI-based neuroimaging

Antibodies

Antibodies used	SMARCB1/BAF47 (D8M1X) Rabbit mAb Cell Signalling 91735 Lot 2<br>Anti-BRD9 antibody Abcam ab137245 Lot GR3372527-6<br>Anti-KMT2D antibody produced in rabbit Sigma HPA035977<br>Anti-Kmt2a MLL1 Antibody Bethyl A300-086A Lot 6<br>IgG Rabbit IgG, polyclonal - Isotype Control (ChIP Grade) 100 ug Abcam ab171870<br>Anti-Stat5a Recombinant Anti-STAT5a antibody [E289] Abcam ab32043 Lot GR3238474-7<br>Anti-Cebpa Abcam ab40764 Lot GR4228581-2<br>Anti-Cebpe Sigma-Aldrich HPA002928<br>Anti-CD45R/B220 BV510 RA3-6B2 BioLegend ref 103247<br>Anti-CD3e BV510 145-2C11 BioLegend ref 100233

Anti-CD11b BV510 M1/70 BioLegend ref 101263
Anti-CD11b PECy7 M1/70 BioLegend ref 101215
Anti-Gr1 BV510 RB6-8C5 BioLegend ref 108437
Anti-Ter119 BV510 Ter-119 BioLegend ref 116237
Anti-CD16/32 (FcgR-III) PercPCy5.5 93 BioLegend ref 101323
Anti-CD34 FITC RAM34 Invitrogen ref 11-0341-82
Anti-CD41 APCCy7 MWReg30 BioLegend ref 133927
Anti-CD45.1 PECy7 A20 BioLegend ref 110730
Anti-CD45.2 APC/Fire750 104 BioLegend ref 109852
Anti-CD55 PE RIKO-3 BioLegend ref 131803
CD117 (c-kit) APC 2B8 BioLegend ref 105812
Anti-Sca1 PE D7 BioLegend ref 108107
Anti-CD11b Total-Seq B M1/70 Biolegend ref 101273
Anti-Ly6C Total-Seq B HK1.4, Biolegend ref 128053
Anti-CD115 Total-Seq B AFS98, Biolegend ref 135543
Anti-CD14 Total-Seq B Sa14-2, Biolegend ref 123341
Anti-CD150 Total-Seq B TC15-12F12.2, Biolegend ref 115951
Anti-CD48 Total-Seq B HM48-1, Biolegend ref 103457
Anti-CD34 Total-Seq B SA376A4, Biolegend ref 152213
Anti-CD117 Total-Seq B 2B8 Biolegend ref 105849
Anti-CD55 Total-Seq B RIKO-3 Biolegend ref 131817
Anti-CD41 Total-Seq B MWReg30 Biolegend ref 133941
Anti-CD326 Total-Seq B G8.8 Biolegend ref 118247
Anti-FceRI Total-Seq B Mar-01 Biolegend ref 134341

Validation

SMARCB1/BAF47 (D8M1X) Rabbit mAb Cell Signalling 91735 Lot 2
Validated by the supplier (Cell Signalling):
Product Usage Information.
For optimal ChIP and ChIP-seq results, use 10 µl of antibody and 10 µg of chromatin (approximately 4 x 106 cells) per IP. This antibody has been validated using SimpleChIP® Enzymatic Chromatin IP Kits.

Anti-BRD9 antibody Abcam ab137245 Lot GR3372527-6
Validation:
1) We compared the binding pattern obtained with this antibody to the Brd9 antibody provided by Active Motif (https://www.activemotif.com/catalog/details/61537/brd9-antibody-pab currently discontinued) and found a strong correlation between both patterns
2) Used for ChIP-seq in the following publication.
Inoue D et al. Spliceosomal disruption of the non-canonical BAF complex in cancer
Nature. 2019 October ; 574(7778): 432–436.

Anti-KMT2D antibody produced in rabbit Sigma HPA035977
Validation:
- Used for ChIP-seq in:
Zhang J et at. Disruption of KMT2D perturbs germinal center B cell development and promotes lymphomagenesis.
Nature Medicine October 2015

Kmt2a MLL1 Antibody Bethyl A300-086A Lot 6
Validation:
- Used for ChIP-seq in 20 publications (see https://www.citeab.com/antibodies/654488-a300-086a-rabbit-anti-mll1-antibody-affinity-purifie) amongst them:
Schwörer, S., et al. Epigenetic stress responses induce muscle stem-cell ageing by Hoxa9 developmental signals.
Nature on 15 December 2016

IgG Rabbit IgG, polyclonal - Isotype Control (ChIP Grade) 100 ug Abcam ab171870
- Validated by the provider (abcam)

Stat5a Recombinant Anti-STAT5a antibody [E289] Abcam ab32043 Lot GR3238474-7
- Used for ChIP-seq in 2 publications
He, L., et al. Local blockage of self-sustainable erythropoietin signaling suppresses tumor progression in non-small cell lung cancer.
Oncotarget on 10 October 2017
Lee, K. M., et al. Inhibition of STAT5A promotes osteogenesis by DLX5 regulation.
Cell Death & Disease on 14 November 2018
https://www.citeab.com/antibodies/775960-ab32043-anti-stat5a-antibody-e289?des=3bc20e5fb5095dd9

Cebpa Abcam ab40764
- Used for ChIP-seq in 4 publications (below are the two most recent)
Qin, Y., Grimm, S. A., et al. Alterations in promoter interaction landscape and transcriptional network underlying metabolic adaptation to diet
Nature Communications on 19 February 2020
Yao, S., Wu, D., et al. Hypermethylation of the G protein-coupled receptor kinase 6 (GRK6) promoter inhibits binding of C/EBPα, and GRK6 knockdown promotes cell migration and invasion in lung adenocarcinoma cells.
FEBS Open Bio on 1 April 2019

Anti-CD45R/B220 BV510 RA3-6B2 BioLegend ref 103247
Validation:

- Used in several publications (see https://www.biolegend.com/ja-jp/products/brilliant-violet-510-anti-mouse-human-cd45r-b220-antibody-7996) amongst them:
Hutter K et al. The miR-15a/16-1 and miR-15b/16-2 clusters regulate early B cell development by limiting IL-7 receptor expression.
Front Immunol. 2022 Aug 25;13:967914.

Anti-CD3e BV510 145-2C11 BioLegend ref 100233
Validation:
- Used in several publications (see https://www.biolegend.com/ja-jp/products/brilliant-violet-510-anti-mouse-cd3-antibody-7990) amongst them:
Shen E et al. Control of Germinal Center Localization and Lineage Stability of Follicular Regulatory T Cells by the Blimp1 Transcription Factor.
Cell Rep. 2019 Nov 12;29(7):1848-1861.e6.

Anti-CD11b BV510 M1/70 BioLegend ref 101263
Validation:
- Used in several publications (see https://www.biolegend.com/ja-jp/products/brilliant-violet-510-anti-mouse-human-cd11b-antibody-7993) amongst them:
Ramakrishna C et al. Bacteroides fragilis polysaccharide A induces IL-10 secreting B and T cells that prevent viral encephalitis.
Nat Commun. 2019 May 14;10(1):2153.

Anti-CD11b PECy7 M1/70 BioLegend ref 101215
Validation:
- Used in several publications (see https://www.biolegend.com/ja-jp/products/pe-cyanine7-anti-mouse-human-cd11b-antibody-1921) amongst them:
Hatzi K et al. Histone demethylase LSD1 is required for germinal center formation and BCL6-driven lymphomagenesis.
Nat Immunol. 2019 Jan;20(1):86-96.

Anti-Gr1 BV510 RB6-8C5 BioLegend ref 108437
Validation:
- Used in several publications (see https://www.biolegend.com/en-us/products/brilliant-violet-510-anti-mouse-ly-6g-ly-6c-gr-1-antibody-8614) amongst them:
Liu Y et al. Rapid acceleration of KRAS-mutant pancreatic carcinogenesis via remodeling of tumor immune microenvironment by PPARδ.
Nat Commun. 2022 May 13;13(1):2665.

Anti-Ter119 BV510 Ter-119 BioLegend ref 116237
Validation:
- Used in several publications (see https://www.biolegend.com/en-us/products/brilliant-violet-510-anti-mouse-ter-119-erythroid-cells-antibody-8243) amongst them:
Yamaguchi A et al. Blockade of the interaction between BMP9 and endoglin on erythroid progenitors promotes erythropoiesis in mice.
Genes Cells. 2021 Oct;26(10):782-797.

Anti-CD16/32 (FcgR-III) PercPCy5.5 93 BioLegend ref 101323
Validation:
- Used in several publications (see https://www.biolegend.com/ja-jp/products/percp-cyanine5-5-anti-mouse-cd16-32-antibody-6165) amongst them:
Viny AD et al. Cohesin Members Stag1 and Stag2 Display Distinct Roles in Chromatin Accessibility and Topological Control of HSC Self-Renewal and Differentiation.
Cell Stem Cell. 2019 Nov 7;25(5):682-696.e8.

Anti-CD34 FITC RAM34 Invitrogen ref 11-0341-82
Validation:
- Used in several publications (see https://www.thermofisher.com/antibody/product/CD34-Antibody-clone-RAM34-Monoclonal/11-0341-82) amongst them:
Wilkinson AC et al. Long-term ex vivo haematopoietic-stem-cell expansion allows nonconditioned transplantation.
Nature. 2019 Jul;571(7763):117-121.

Anti-CD41 APCCy7 MWReg30 BioLegend ref 133927
Validation:
- Used in several publications (see https://www.biolegend.com/ja-jp/products/apc-cyanine7-anti-mouse-cd41-antibody-13014) amongst them:
Al-Rifai R et al. JAK2V617F mutation drives vascular resident macrophages toward a pathogenic phenotype and promotes dissecting aortic aneurysm.
Nat Commun. 2022 Nov 3;13(1):6592.

Anti-CD45.1 PECy7 A20 BioLegend ref 110730
Validation:
- Used in several publications (see https://www.biolegend.com/ja-jp/products/pe-cyanine7-anti-mouse-cd45-1-antibody-4917) amongst them:
Garo LP et al. MicroRNA-146a limits tumorigenic inflammation in colorectal cancer.
Nat Commun. 2021 Apr 23;12(1):2419.

Anti-CD45.2 APC/Fire750 104 BioLegend ref 109852
Validation:
- Used in several publications (see https://www.biolegend.com/ja-jp/products/apc-fire-750-anti-mouse-cd45-2-antibody-13589)

amongst them:
Formaglio P et al. Nitric oxide controls proliferation of Leishmania major by inhibiting the recruitment of permissive host cells. Immunity. 2021 Dec 14;54(12):2724-2739.e10.

Anti-CD55 PE RIKO-3 BioLegend ref 131803
Validation:
- Used in several publications (see https://www.biolegend.com/ja-jp/products/pe-anti-mouse-cd55-daf-antibody-5514) amongst them:
Camps J et al. Interstitial Cell Remodeling Promotes Aberrant Adipogenesis in Dystrophic Muscles.
Cell Rep. 2020 May 5;31(5):107597.

CD117 (c-kit) APC 2B8 BioLegend ref 105812
Validation:
- Used in several publications (see https://www.biolegend.com/ja-jp/products/apc-anti-mouse-cd117-c-kit-antibody-72) amongst them:
Lawson H et al. CITED2 coordinates key hematopoietic regulatory pathways to maintain the HSC pool in both steady-state hematopoiesis and transplantation.
Stem Cell Reports. 2021 Nov 9;16(11):2784-2797.

Anti-Sca1 PE D7 BioLegend ref 108107
Validation:
- Used in several publications (see https://www.biolegend.com/ja-jp/products/pe-anti-mouse-ly-6a-e-sca-1-antibody-228) amongst them:
Tran NT et al. Efficient CRISPR/Cas9-Mediated Gene Knockin in Mouse Hematopoietic Stem and Progenitor Cells.
Cell Rep. 2019 Sep 24;28(13):3510-3522.e5.

Eukaryotic cell lines

Policy information about cell lines and Sex and Gender in Research

Cell line source(s): HEK 293T (Sigma, 12022001-DNA-5UG)

Authentication: Purchased from the provider (Sigma) as an autheticated cell line. We did not performed any further authetication and used early passages (p< 8) were used for lentivirus production.

Mycoplasma contamination: Cell lines were tested negative for Mycoplasma

Commonly misidentified lines (See ICLAC register): No commonly misidentified cells were used

Animals and other research organisms

Policy information about studies involving animals; ARRIVE guidelines recommended for reporting animal research, and Sex and Gender in Research

Laboratory animals

C57BL/6J (Jackson Laboratory #JAX_000664)
Age: 12-14 weeks
Sex: Equal numbers of males and females

Gt(ROSA)26Sortm1.1(CAG-cas9*/EGFP)Rsky (Jackson Laboratory #JAX_026179)
Age: 12-15 weeks
Sex: Equal numbers of males and females

B6.SJL-Ptprca Pepcb/BoyJ (CD45.1) (Jackson #002014)
Age: 12 weeks
Sex: Equal numbers of males and females

Npm1c/Flt3ITD/Cas9 (Huntly lab) - Primary leukemic cells obtained from bone-marrow tumours were derived from this strain.
Age: 12 weeks
Sex: Female

Murine ethical compliance was fulfilled under the Guidelines of the Care and Use of Laboratory Animals and were approved by the Institutional Animal Care and Use Committees at University of Navarra, Spain, and the Animal Welfare Ethical Review Body at the University of Cambridge, UK. Research in the UK was conducted under Home Office license PP3042348.

Wild animals: The study does not involve wild animals

Reporting on sex

Equal numbers of female and males were used to:
- Obtain Haematopoietic progenitors for Bulk CRISPR screens and Perturb-seq experiments
- Isolate cell populations for ChIP-seq

Field-collected samples: The study does not involve field-collected samples

Ethics oversight	All animal procedures were completed in accordance with the Guidelines of the Care and Use of Laboratory Animals and were approved by the Institutional Animal Care and Use Committees at University of Navarra, Spain, and the Animal Welfare Ethical Review Body at the University of Cambridge, UK.

Note that full information on the approval of the study protocol must also be provided in the manuscript.

Methodology

Replicates

Every ChIP seq analysis was performed with two replicate independent ChIP-seq experiments except for Kmt2a and Kmt2d ChIP-seq in myeloid and erythroid progenitors, where due to the difficulty of getting enough cells numbers the analysis of Brd9 and Smarcb1 ChIP-seq patterns was prioritised.

Sequencing depth

GEO name	Total Reads	Aligned reads after trimming, mapping, removing duplicates and blacklisted peeaks
Kmt2a-DM_rep1	71556716	52135927
Kmt2a-DM_rep2	20458692	16266441
Kmt2a-exvivoMonocytes_rep1	69985617	41947055
Kmt2a-exvivoMonocytes_rep2	12682223	9845731
Kmt2a-Ery	82954531	56071969
Kmt2a-GMP	73167956	53421804
Kmt2a-MEP	97640262	70833084
Kmt2a-Bcell	67491226	44742639
Kmt2a-Monocytes_rep1	32369273	25607927
Kmt2a-Monocytes_rep2	34539498	26022063
Kmt2d-exvivoMonocytes_rep1	41825984	26001792
Kmt2d-exvivoMonocytes_rep2	44949940	24358640
Kmt2d-exvivoMonocytes_rep3	72158751	48488577
Kmt2d-DM_rep1	25995989	16107654
Kmt2d-DM_rep2	90366626	59794611
Kmt2d-GMP	116393335	63951963
Kmt2d-MEP	43313559	65504507
Kmt2d-Ery_rep1	35064637	20413963
Kmt2d-Ery_rep2	37652595	22496310
Kmt2d-Bcell	38868015	23667460
Kmt2d-Monocytes_rep1	27979280	19401634
Kmt2d-Monocytes_rep2	23938072	16373041
Smarcb1-exvivoMonocytes_rep1	52835039	32356601
Smarcb1-exvivoMonocytes_rep2	41122100	29455975
Smarcb1-exvivoMonocytes_rep3	44557784	27003578
Smarcb1-DM_rep1	42890145	28328521
Smarcb1-DM_rep2	104296115	75890008
Smarcb1-GMP	92342249	58562130
Smarcb1-MEP	46733792	24150364
Smarcb1-Monocytes_rep1	20789867	14779534
Smarcb1-Monocytes_rep2	26354572	18880512
Smarcb1-Ery	18975965	13336679
Brd9-DM_rep1	65031476	43919123
Brd9-DM_rep2	95350112	67667153
Brd9-exvivoMonocytes	27240753	19317290
Brd9-GMP_rep1	52193534	36246200
Brd9-GMP_rep2	23607799	16429228
Brd9-MEP_rep1	62518967	46671329
Brd9-MEP_rep2	24865443	18494644
Brd9-MEP_rep3	40688029	28876910
Brd9-Ery_rep1	17103452	11946664
Brd9-Ery_rep2	24144295	16847147
Brd9-Monocytes_rep1	26536687	18426771
Brd9-Monocytes_rep2	26333906	19067019
Brd9-Bcell	14051972	9065470
Stat5a-DM	63783822	30893003
Stat5a-exvivoMonocytes	54541020	23981871

Antibodies

SMARCB1/BAF47 (D8M1X) Rabbit mAb Cell Signalling 91735 Lot 2
Anti-BRD9 antibody Abcam ab137245 Lot GR3372527-6
Anti-KMT2D antibody produced in rabbit Sigma HPA035977
Kmt2a MLL1 Antibody Bethyl A300-086A Lot 6
IgG Rabbit IgG, polyclonal - Isotype Control (ChIP Grade) 100 ug Abcam ab171870
Stat5a Recombinant Anti-STAT5a antibody [E289] Abcam ab32043 Lot GR3238474-7
Runx1 abcam ab23980 lot GR3213439-2
Runx2 abcam ab236639 lot GR3388032-15
Cebpa Abcam ab40764
CEBPE Sigma-Aldrich HPA002928

Peak calling parameters

ChIP-seq reads were aligned to the GRCm38/mm10 reference genome assembly using Bowtie version 2.3.4.2 with parameters -X 1000 --no-discordant --no-mixed --very-sensitive. Peaks were called using MACS v2.2.7.1 with parameters -f BAMPE--keep-dup all and IgG as control.

Data quality

We followed the ChIP-seq nf-core pipeline. ChIP-seq reads were trimmed with default parameters using Trim Galore with Cutadapt. ChIP-seq reads were aligned to the GRCm38/mm10 reference genome assembly using Bowtie with parameters -X 1000 --no-discordant --no-mixed --very-sensitive.

ChIP-seq

Data deposition

☒ Confirm that both raw and final processed data have been deposited in a public database such as GEO.

☒ Confirm that you have deposited or provided access to graph files (e.g. BED files) for the called peaks.

Data access links<br>*May remain private before publication.*	ChIP-seq datasets of CFs in vivo, ex vivo and leukaemic: GEO accession (GSE213507)<br>To review GEO accession GSE213513:<br>Go to https://www.ncbi.nlm.nih.gov/geo/query/acc.cgi?acc=GSE213513<br>Enter token whufyuosxjirrgx into the box
Files in database submission	GSM6588342 Smarcb1-GMP<br>GSM6588343 Smarcb1-MEP<br>GSM6588344 Smarcb1-Ery<br>GSM6588345 Smarcb1-Monocytes_rep1<br>GSM6588346 Smarcb1-Monocytes_rep2<br>GSM6588347 Smarcb1-exvivoMonocytes_rep1<br>GSM6588348 Smarcb1-exvivoMonocytes_rep2<br>GSM6588349 Smarcb1-exvivoMonocytes_rep3<br>GSM6588350 Smarcb1-DM_rep1<br>GSM6588351 Smarcb1-DM_rep2<br>GSM6588352 Brd9-GMP_rep1<br>GSM6588353 Brd9-GMP_rep2<br>GSM6588354 Brd9-MEP_rep1<br>GSM6588355 Brd9-MEP_rep2<br>GSM6588356 Brd9-MEP_rep3<br>GSM6588357 Brd9-Ery_rep1<br>GSM6588358 Brd9-Ery_rep2<br>GSM6588359 Brd9-Bcell<br>GSM6588360 Brd9-Monocytes_rep1<br>GSM6588361 Brd9-Monocytes_rep2<br>GSM6588362 Brd9-exvivoMonocytes<br>GSM6588363 Brd9-DM_rep1<br>GSM6588364 Brd9-DM_rep2<br>GSM6588365 Kmt2d-GMP<br>GSM6588366 Kmt2d-MEP<br>GSM6588367 Kmt2d-Ery_rep1<br>GSM6588368 Kmt2d-Ery_rep2<br>GSM6588369 Kmt2d-Bcell<br>GSM6588370 Kmt2d-Monocytes_rep1<br>GSM6588371 Kmt2d-Monocytes_rep2<br>GSM6588372 Kmt2d-exvivoMonocytes_rep1<br>GSM6588373 Kmt2d-exvivoMonocytes_rep2<br>GSM6588374 Kmt2d-exvivoMonocytes_rep3<br>GSM6588375 Kmt2d-DM_rep1<br>GSM6588376 Kmt2d-DM_rep2<br>GSM6588377 Kmt2a-GMP<br>GSM6588378 Kmt2a-MEP<br>GSM6588379 Kmt2a-Ery<br>GSM6588380 Kmt2a-Bcell<br>GSM6588381 Kmt2a-Monocytes_rep1<br>GSM6588382 Kmt2a-Monocytes_rep2<br>GSM6588383 Kmt2a-exvivoMonocytes_rep1<br>GSM6588384 Kmt2a-exvivoMonocytes_rep2<br>GSM6588385 Kmt2a-DM_rep1<br>GSM6588386 Kmt2a-DM_rep2<br>GSM6588387 Stat5a-exvivoMonocytes<br>GSM6588388 Stat5a-DM<br>GSM6588389 IgG-DM<br>GSM6588390 IgG-exvivoMonocytes_rep1<br>GSM6588391 IgG-exvivoMonocytes_rep2
Genome browser session<br>(e.g. UCSC)	https://genome.ucsc.edu/s/julenm/Lara-Astiaso_et_al

We removed duplicated regions with Picard Tools and filtered out ENCODE blacklist regions and non-interesting chromosomes.
We pooled replicates.
Peaks were called using MACS with parameters -f BAMPE--keep-dup all.
Peaks at FDR 5% and above 5-fold enrichment vs IgG:

GMP_Smarcb1_ChIP11_peaks.narrowPeak 17325
MEP_Smarcb1_ChIP12_peaks.narrowPeak 5453
Leukaemia_Smarcb1_peaks.narrowPeak 15777

Exvivo-Mono_Smarcb1_peaks.narrowPeak 16333
Mono_Smarcb1_peaks.narrowPeak 11194
Ery_Smarcb1_peaks.narrowPeak 30

GMP_Brd9_peaks.narrowPeak 5124
MEP_Brd9_peaks.narrowPeak 8371
Exvivo-Mono_Brd9_peaks.narrowPeak 11648
Mono_Brd9_peaks.narrowPeak 11719
Bcell_Brd9_peaks.narrowPeak 3118
Ery_Brd9_peaks.narrowPeak 2168

GMP_Kmt2d_peaks.narrowPeak 22114
MEP_Kmt2d_peaks.narrowPeak 3111
Leukaemia_Kmt2d_peaks.narrowPeak 29038
Exvivo-Mono_Kmt2d_peaks.narrowPeak 18123
Mono_Kmt2d-merged_peaks.narrowPeak 7567
Bcell_Kmt2d_peaks.narrowPeak 8285
Ery_Kmt2d_peaks.narrowPeak 1266

GMP_Kmt2a_peaks.narrowPeak 33869
MEP_Kmt2a_peaks.narrowPeak 28581
Leukaemia_Kmt2a_peaks.narrowPeak 17532
Exvivo-Mono_Kmt2a_peaks.narrowPeak 12585
Mono_Kmt2a_peaks.narrowPeak 1346
Ery_Kmt2a_peaks.narrowPeak 5413
Bcell_Kmt2a_peaks.narrowPeak 13741

Leukaemia_Stat5a_peaks.narrowPeak 26878
Exvivo-Mono_Stat5a_peaks.narrowPeak 16857

Software

Trim Galore v0.6.6
Cutadapt v3.4
Bowtie v2.3.4.2
Picard v2.25.4
ENCODE blacklist regions v2.0
MACS v2.2.7.1
Code is available at https://github.com/csbg/tfcf/tree/main/ATAC_ChIP/ChIP

Flow Cytometry

Plots

Confirm that:

☒ The axis labels state the marker and fluorochrome used (e.g. CD4-FITC).

☒ The axis scales are clearly visible. Include numbers along axes only for bottom left plot of group (a 'group' is an analysis of identical markers).

☒ All plots are contour plots with outliers or pseudocolor plots.

☒ A numerical value for number of cells or percentage (with statistics) is provided.

Methodology

Sample preparation

Sorting of haematopoietic progenitors for Exvivo CRISPR Screens
Femora, tibiae, ilia, humerus, sternum and scapula were harvested from 12-14 week old C57BL/6J and ROSAxCas9 mice (equal ratio of males and females), crushed with a pestle and mortar using cold (4 ºC) autoMACS Running Buffer and filtered through a 70 µM strainer. Red Blood Cells were lysed using RBC Lysis Buffer and c-Kit+ cells were enriched using mouse CD117 magnetic beads (Miltenyi) , following the manufacturer’s protocol. The c-Kit enriched fraction was stained with anti-Lineage (B220, CD3, CD11b, Gr1, Ter-119), anti-CD117 (cKit) and PE anti-Sca1. Lin-/cKit+/Sca1+ hematopoietic progenitors cells (LSKs) were FACS-sorted in 1 mL of DMEM/F12 + 1X Pen/Strep.

Ex vivo CRISPR FACS Readouts.
Cultures were harvested by centrifugation at 300 g for 5 minutes and washed twice with 1X cold PBS. Then the cell pellets were stained with the Readout specific cocktails (see below) plus a viability marker (TOPRO or Propidium Iodide). Viable BFP+ cells (containing CRISPR guides) were gated from Cas9 (GFP+) and Non-Cas9 (GFP-) fractions and, from each fraction the readout populations (see below) were sorted in 1.5 mL tubes containing PBS + 0.1% BSA.

In vivo CRISPR Screens
Femora, tibiae, ilia, humerus, sternum and scapula were harvested from bone marrow transplanted B6.SJL-Ptprca Pepcb/BoyJ (CD45.1), crushed with a pestle and mortar using cold (4 ºC) autoMACS Running Buffer and filtered through a 70 µM

strainer. Red Blood Cells were lysed using RBC Lysis Buffer and c-Kit+ cells were enriched using mouse CD117 magnetic beads (Miltenyi) , following the manufacturer's protocol. The purified cKit+ fraction was stained with TOPRO (viability), anti-Lineage (CD3, CD19, Ter119, CD11b, Gr1) and anti-CD117 (cKit) antibodies. For single-cell RNAseq we FACS-sorted 200,000 viable (TOPRO-), GFP+ (Cas9), BFP+ (sgRNA) cells from Lineage- and Lineage+/cKit+ fractions and processed each of them in a 10X single-cell RNA-seq partition aiming at a final coverage of 500 single-cells per sgRNA.

Isolation of in vivo hematopoietic cells for ChIP-seq.
Murine haematopoietic cells were harvested from 12-14 week old C57BL6 mice (balanced numbers of males and females) as described above and stained for the isolation of:
GMP: Lineage (CD3, CD19, CD11b, Gr1, Ter119, B220)-, cKit+, Sca-1-, FcgRIII+, CD34+
MEP: Lineage (CD3, CD19, CD11b, Gr1, Ter119, B220)-, cKit+, Sca-1-, FcgRIII-, CD34-
Monocytes: CD3-, CD19-, Ter119-, CD11b+
B-cell: CD3-, CD19+, Ter119-, CD11b-
Erythroid cells were FACS-sorted from spleens of 12 week-old C57BL6 mice as: CD3-, CD19-, Ter119+, CD11b-, Gr1-.
Cells were sorted in PBS + 0.1% BSA and crosslinked immediately after sorting

Analysis of leukaemic populations exvivo.
Npm1c/Flt3-ITD/Cas9 double mutant (DM) cells were generated from lineage-depleted, bone marrow cells of primary transgenic mice post-leukemic onset. Cells were maintained in XVIVO-20 medium (Lonza) supplemented with 5% Fetal Bovine Serum (FBS) (ThermoFisherScientific), 1% PSG (Gibco), murine SCF 50 ng/mL (PeproTech), murine IL-3 10 ng/mL (PeproTech) and murine IL-6 10 ng/mL (R&D Systems), in a 37oC and 5% CO2 atmospheric environment. Npm1c/Flt3-ITD/Cas9 double mutant (DM) cells were passaged every 2 days and cultured for short time (passage 3-5) to maintain the original leukaemic properties.
For FACS analysis, cells were washed twce with ice-cold PBS and stained with:
CD11b (PE-Cy7 conjugated; clone M1/70; BD Biosciences),
Gr-1 (Ly6G/Ly6C; APC-Cy7 conjugated; RB6-8C5 clone; BD Biosciences),
CD55 (PE-conjugated; clone RIKO-3; Biolegend),
CD41 (APC-conjugated; clone MWReg30; Biolegend) and
CD34 (FITC-conjugated; clone RAM34; BD Bioscience).
Gran-like (CD11b-high/Gr-1+); Ery/Baso-like (CD55-high/CD41-) and CD34+ fractions were subsequently FACS-sorted (BD Influx; BD Bioscience)

Instrument

BD LSR Fortessa II; BD Biosciences
BD Influx; BD Bioscience

Software

FlowJo (version 10.8.0)

Cell population abundance

The purity of the post sorting fractions was further characterized with scRNA-seq

Gating strategy

LSK purificaton
1- Exclude doublets
2- Remove debris
3- Gate Lineage-negative cells
4- Gate ckit-positive, Sca1-positive

FACS Readouts in CRISPR screens (Progenitor vs Differentated):
1- Exclude doublets
2- Remove debris
3- Gate GFP-positive (Cas9), BFP-ckit-positive, Sca1-positive
4- Gate Lineage-negative cells
5- Gate:
a) ckit-positive, Sca1-positive = Multipotent Progenitors
b) ckit-positive, Sca1-negative = Differentiated

FACS Readouts in CRISPR screens (Myeloid vs Mega-erythroid)
1- Exclude doublets
2- Remove debris
3- Gate GFP-positive (Cas9), BFP-ckit-positive, Sca1-positive
4- Gate Lineage-negative cells
5- Gate ckit-positive, Sca1-negative
6- Gate:
a) FcgR-III positive = Myeloid progenitors
b) FcgR-III negative = Mega-erythroid progenitors

FACS Readouts in CRISPR screens (Myeloid vs non-myeloid)
1- Exclude doublets
2- Remove debris
3- Gate GFP-positive (Cas9), BFP-positive (sgRNA)
4- Gate:
a) FcgR-III positive, CD11b-postive = Myeloid fraction
b) FcgR-III negative, CD11b-negative = Non-myeloid

FACS Readouts in CRISPR screens (Terminal myeloid differentiation)
1- Exclude doublets

2- Remove debris
3- Gate GFP-positive (Cas9), BFP-positive (sgRNA)
4- Gate:
a) Gr1-positive, CD11b-postive = Mature Myeloid cells
b) Gr1-negative, CD11b-negative = Myeloid progenitors

FACS-sorting for in vivo Perturb-seq
1- Exclude doublets
2- Gate viable cells
3- Gate BFP-positive (CRISPRed cells)
4- Gate:
a) Lineage-negative= Lin-negative fraction
b) Lineage-positive, ckt-positive= Lin-neg/ckit-pos fraction

FACS-sorting for isolation of leukemic cells
1- Exclude doublets
2- Gate viable cells
3- Gate Lineage-negative cells

FACS-sorting for Perturb-seq in Leukemia
1- Exclude doublets
2- Gate viable cells
3- Gate BFP-positive (CRISPRed cells)

Exemplar FACS plots can be found in the Supplementary Materials

☒ Tick this box to confirm that a figure exemplifying the gating strategy is provided in the Supplementary Information.
